# Measurement of Urinary Bladder Pressure: A Comparison of Methods

**DOI:** 10.3390/s18072128

**Published:** 2018-07-03

**Authors:** Ingelin Clausen, Lars Geir W. Tvedt, Thomas Glott

**Affiliations:** 1 Department of Microsystems and Nanotechnology, SINTEF Digital, NO-0314 Oslo, Norway; lars.g.w.tvedt@sintef.no; 2 Sunnaas Rehabilitation Hospital HF, NO-1450 Nesoddtangen, Norway; thomas.glott@sunnaas.no

**Keywords:** in vivo pressure, pressure sensor, MEMS, cystometry

## Abstract

Pressure is an essential parameter for the normal function of almost all organs in the human body. Measurement of pressure is therefore highly important in clinical practice and medical research. In clinical practice, pressures are often measured indirectly through a fluid line where the pressure is transmitted from the organ of interest to a remote, externally localized transducer. This method has several limitations and is prone to artefacts from movements. Results from an in vitro bench study comparing the characteristics of two different sensor systems for bladder assessment are presented; a new cystometry system using a MEMS-based in-target organ sensor was compared with a conventional system using water-filled lines connected to external transducers. Robustness to measurement errors due to patient movement was investigated through response to forced vibrations. While the new cystometry system detected real changes in applied pressure for excitation frequencies ranging from 5 Hz to 25 Hz, such small and high-frequency stimuli were not transmitted through the water-filled line connected to the external transducer. The new sensor system worked well after a resilient test at frequencies up to 70 Hz. The in-target organ sensor system will offer new possibilities for long-term monitoring of in vivo pressure in general. This opens up the possibility for future personalized medical treatment and renders possible new health services and, thereby, an increased patient empowerment and quality of life.

## 1. Introduction

Pressure is an essential parameter for the normal function of almost all organs in the human body. Deviation of pressure out of range for physiological function may result in injury or deteriorating organ function. As such, measurement of pressure is highly important in clinical practice and medical research. Pressures in the circulatory system, intraocular, urinary bladder, gastrointestinal tract, muscle compartments, and joints are some examples of pressures being routinely measured. Although the target organ may have a “slow physiological response”, any patient movement may influence measurements significantly. Therefore, the most precise measurements are currently done invasively and stationary, that is, with the patient supine. Examples are measurements of cardiac-, intracranial-, and bladder- pressures.

In clinical practice, pressures are often measured indirectly through a fluid line where pressure is transmitted from the organ of interest to a remote, externally localized transducer. Transmission through a fluid line represents a delay in the measurements. Pressure measurement through a fluid column is also prone to artefacts from patient movement. Both due to patient comfort and risk of infections, the pressure examination is mostly limited to a short period of time, for example, during or after surgery, and only instantaneous values are recorded. Therefore, conventional pressure measurement methods represent only the best achievable measure of the patient’s condition.

To reveal the exact organ condition, continuous organ monitoring ideally lasting for days, weeks, or years is needed. Such monitoring allows for not only real-time measurements, but also for personalized medical treatment. Combined with alarms being activated when measured values exceed a certain limit, organ monitoring on a continuous base can render possible new health services and thereby increase patient empowerment and the quality of life. One premise is that the patient must have the possibility to move and keep up normal activities during the measurement period, that is, the measurement method should not be vulnerable to patient movements or other artefacts. Small-sized, lightweight, and energy-efficient Micro Electro Mechanical Systems (MEMS) combined with a minimally invasive method may represent a solution to this. Although research on permanently implantable sensors for the monitoring of bodily pressure has been conducted for decades [1,2,3], more research is still needed. Immunologic responses to the implant size, structure, and material, combined with individual immune systems, represent serious hindrances for the success. In vivo pressure measurements are particularly demanding, as contact (direct or indirect) between the pressurizing medium and the sensor is required. In general, any foreign implant is at risk of being encapsulated in fibrous tissue, or “scar tissue”. For an implanted sensor, sensor drift and, in the end, sensor failure might be the result. Additionally, corrosion caused by aggressive body fluids may alter the sensor characteristics and thereby sensor stability and again result in sensor failure. To our knowledge, only one implantable pressure sensor for long-term use has been launched in the market [4].

One patient group that can benefit from continuous organ monitoring, with or without alarm capabilities, is those who are suffering from a dysfunctional urinary bladder. Voiding dysfunction, for example, urgency and incontinence, are common symptoms in the general population. Urinary incontinence occurs in approximately 10% of the male population [5]. In a Norwegian study, approximately one out of four reported some problems with incontinence [6]. The severity varied, but as many as 7% reported incontinence as a considerable problem. The consequences can be disabling medically, physically, and socially. There are several options available for treating these consequences like medication, assistive devices, or surgery. The management of voiding dysfunction requires careful assessment of how the bladder is responding during filling and emptying, and recording bladder pressure is essential. Measurement of bladder pressure, in medical terms known as cystometry, is the method by which the storage function of the lower urinary tract is measured during the filling of the bladder. Cystometry is, in general, regarded as the “golden standard” for assessment of lower urinary tract dysfunction by the International Continence Society (ICS) [7]. However, there is a large variation in the clinical use of cystometric examinations. Access to equipment, financial resources, and existing routines in clinical practice are important factors. Based on the prevalence in Norway [5], and expected to be also valid for other western countries, there is a need for about 2000 examinations per million inhabitants annually.

**Figure 1:**
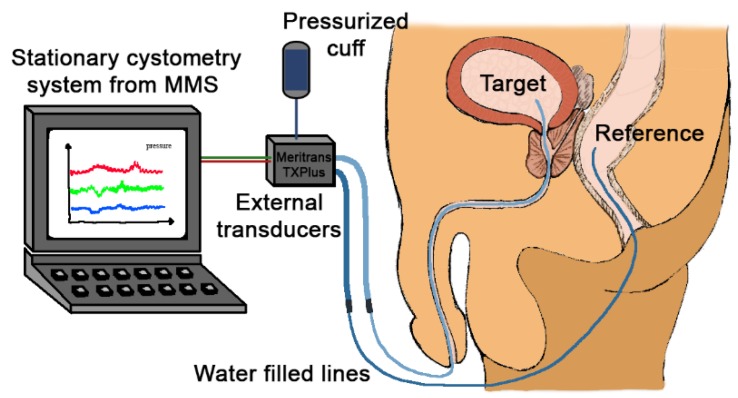
The illustration of the conventional cystometric examination. The setup illustrated here is used for the experimental set-up on the artificial bladder presented in this paper.

The cystometric procedure requires a specialized laboratory and is performed by skilled health personnel. The conventional method involves placement of a catheter through the urethra into the bladder, with a transducer in the distal end. The reference pressure is usually recorded in the rectum. Both target pressure and reference pressure are recorded through water-filled lines (Figure 1). In conventional cystometry, the patients will need to sit or lie down in a laboratory, and the bladder is artificially filled using a pump. The conventional cystometry is done with non-physiological filling rates and urethra obstructed by a catheter. It is also prone to artefacts caused by movements and measurement errors [8].

Through a multidisciplinary approach, SINTEF Digital and Sunnaas Rehabilitation Hospital have over the past five years developed a new in-target organ pressure sensor system for cystometry [9,10]. The new sensor system is based on Micro Electro Mechanical Systems (MEMS) technology and allows for a suprapubic application of sensors in an ambulatory patient.

In this paper, we present results from an in vitro bench study comparing the characteristics of the new MEMS-based sensor system with a conventional system using water-filled lines. Robustness to measurement errors due to movements was investigated through response to forced vibrations, as a superior response to rapid pressure changes will improve the quality of a recording.

## 2. Materials and Methods

### 2.1. The Artificial Bladder Model

In the presented in vitro test, the human urinary bladder was replaced by an artificial flexible bag of latex, filled with approximately 100 mL water. For convenience, the water-filled bag represented both the target organ and the reference body part.

### 2.2. The MEMS-Based Sensor System

**Figure 2:**
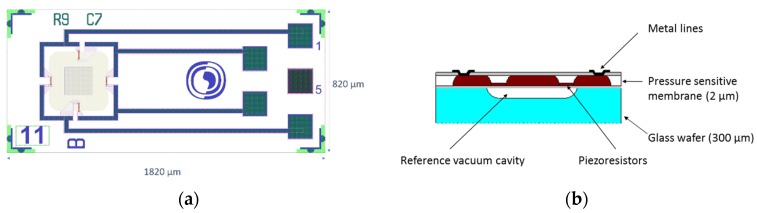
(**a**) A MEMS sensor element, top side view; (**b**) cross-sectional view.

The core component in the new sensor system is a single-crystal MEMS sensor element developed by SINTEF and specially designed for in vivo applications with respect to size and to protection towards bodily fluids. Pressure is determined by deflection of a Si diaphragm comprising four p-type piezoresistors in a full Wheatstone bridge configuration (Figure 2a). The piezoresistors are placed at the diaphragm surface facing a vacuum reference cavity, thus, not in contact with the harsh bodily environment (Figure 2b).

**Figure 3:**
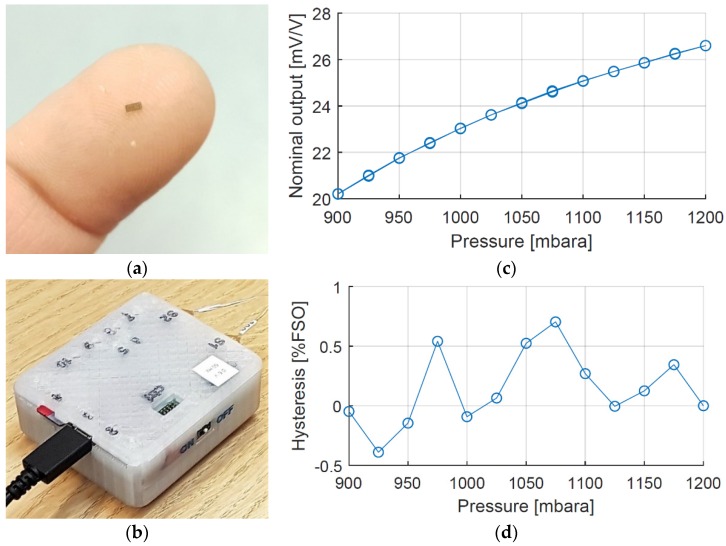
(**a**) A MEMS sensor element designed for in vivo applications; (**b**) Custom-made Sensor Data Logger (SDL) with two sensor probes connected; (**c**) A typical calibration curve from 900 to 1200 mbar absolute pressure (mbara); (**d**) Hysteresis (lower right) of the MEMS sensor for the range from 900 to 1200 mbar, absolute pressure (mbara).

With a piezoresistive principle of measurement, the required electronics can be separated from the sensor element and placed at a location where the space restriction is less severe, here externally on the body. Consequently, the size of the sensor element can be kept small (Figure 3a). The outer lateral dimensions are 820 µm × 1820 µm and the sensor element is integrated into a sensor probe with an outer diameter of only 1.2 mm. All probe materials comply with biocompatibility standards (USP Class VI and/or ISO-10993), but for confidentiality reasons, further details are not given here. More details regarding design, fabrication process, performance, and results from tests of biocompatibility can be found in published papers [11,12]. The sensor bias voltage and the sensor signal voltage are both handled by a custom-made portable external electronic Sensor Data Logger (SDL) consisting of ADCs, a microcontroller, batteries, and a micro SD card for storing the measurement data (Figure 3b). Conversion to mbar is done using a calibration curve of the specific MEMS sensor probe used. A typical calibration curve of the MEMS sensor is shown in Figure 3c, together with the hysteresis in Figure 3d. The MEMS-based sensor probe measures in the entire range of interest, for cystometry typically 0–150 cm H_2_O (medically used measure) corresponding to 1000–1150 mbar absolute pressure (mbara) in engineering terms. As can be seen from Figure 3c,b, the sensitivity is about 25 (µV/V)/mbar and the hysteresis is typically within 5% of full-scale output (%FSO).

**Figure 4:**
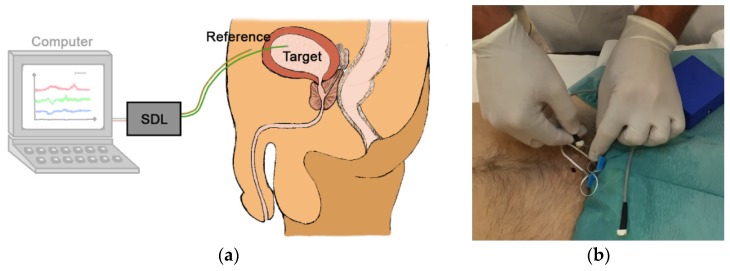
(**a**) The illustration of the suprapubic approach with one target sensor probe in the urinary bladder and a reference sensor probe outside the bladder; (**b**) Suprapubic approach for cystometry, used in a clinical trial on a sample of convenience.

The MEMS-based system has been applied in a suprapubic approach for cystometry (Figure 4). The solution is not yet commercially available, but a clinical trial on a sample of convenience is currently carried out [9].

### 2.3. The Comparator

As a comparator, we used a conventional, stationary cystometry system from Medical Measurements Systems (MMS), (Enschede, The Netherlands), now being part of Laborie, (Mississauga, ON, Canada), [13]. We applied a 200 cm long connection line from Mediplus (Mediplus 9200 extension set 200 cm long, Unit 7) connected to bladder pressure catheters (4.5Fr Mediplus 5100) (Mediplus, High Wycombe, UK), as provided for clinical use by the supplier. To reflect a realistic measurement environment we used two lines, one for the target pressure and another for the reference pressure. The lines were filled with sterile water and connected to the external transducers Meritrans® DTXPlus (Merit Medical Singapore Pte Ltd, Singapore). Filling of all the lines was done by pressurized cuff (inflated to 300 mm Hg) around a bag with 1000 mL sterile water. Perfusion rate through the connection line and bladder pressure catheters was approximately 2–3 mL/min. Repeated flushing of the lines was done to ensure a continuous water column with no air bubbles or other filling defects. We used a sampling rate of 50 Hz and not the standard sampling of 8 Hz commonly used in cystometry. Except for the sampling rate, the system setup was analogue to a routine cystometry examination.

### 2.4. The Experimental Set-Up

**Figure 5:**
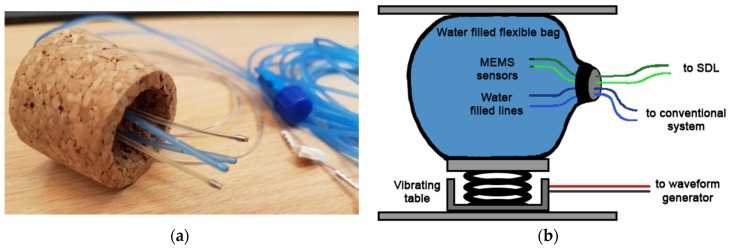
(**a**) The MEMS sensor probes and conventional bladder pressure catheters inserted through the cork to be assembled into the water-filled flexible bag. (**b**) Schematics of the experimental setup.

Two MEMS sensor probes and two bladder pressure catheters were inserted into the flexible bag. One MEMS sensor probe and one pressure catheter represented the pressure sensor and the pressure catheter placed inside the bladder, and the other MEMS sensor probe and pressure catheter represented the reference sensor. The MEMS sensor probes and the bladder pressure catheters were thread through a cork cap and positioned in the neck of the water-filled bag (Figure 5a). The MEMS sensor probes were connected to the external Sensor Data Logger (SDL), while the fluid lines were connected to the conventional, stationary cystometry system. The water-filled bag was placed on a vibrating table (Aura Bass Shaker). The water-filled bag was then fixated in between the shaker and a fixed top plate, to ensure that the vibrations from the shaker were well transferred into the water of the bag (Figure 5b).

The excitation signal for the shaker was controlled by an Agilent 33500B Waveform Generator (Agilent Technologies, Santa Clara, CA, USA) and amplified using a Roxcore A32 Integrated Amplifier (Roxcore, Malmö, Sweden). We then imposed vibrations of frequencies 5 Hz, 10 Hz, and 25 Hz to the shaker. To the author’s knowledge, clinically relevant limits for vibration testing are not established and the frequencies were based on convenience. Water pressure measurements were recorded for 3 min at each excitation frequency applying both the conventional method and the new MEMS-based sensor system. For the MEMS-based sensor system, we sampled with a rate of 50 Hz. We also forced vibrations of 50 Hz and 70 Hz for 3 min to the new system. However, for these high frequencies, the intention was not to perform any pressure measurements, but rather to make an extra resilient test. Normal functioning of the conventional cystometry equipment was verified by testing for pressure increase above zero after completing the vibration tests.

The output readings from the SDL were in mV/V and not converted to mbar, which is sufficient for the purpose of analyzing the sensor response to the high-frequency pressure excitations. The frequency spectra of the data sets were analyzed by the fast Fourier transform (fft) algorithm in MATLAB [14].

## 3. Results

### 3.1. The MEMS-Based in-Target Organ Sensor System

**Figure 6:**
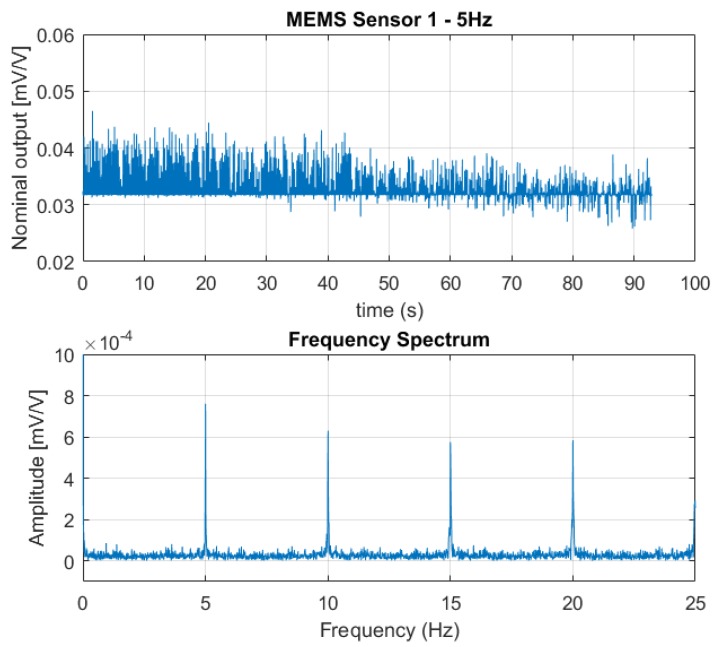
The output signal from one of the sensors in the MEMS-based sensor system as a response to the 5 Hz excitation vibration (**above**) together with fast Fourier transform of the data set (**below**). The fast Fourier Transform clearly shows the fundamental excitation frequency at 5 Hz with an amplitude far above the noise floor; also, the higher order harmonics at 10 Hz, 15 Hz, and 20 Hz are visible.

**Figure 7:**
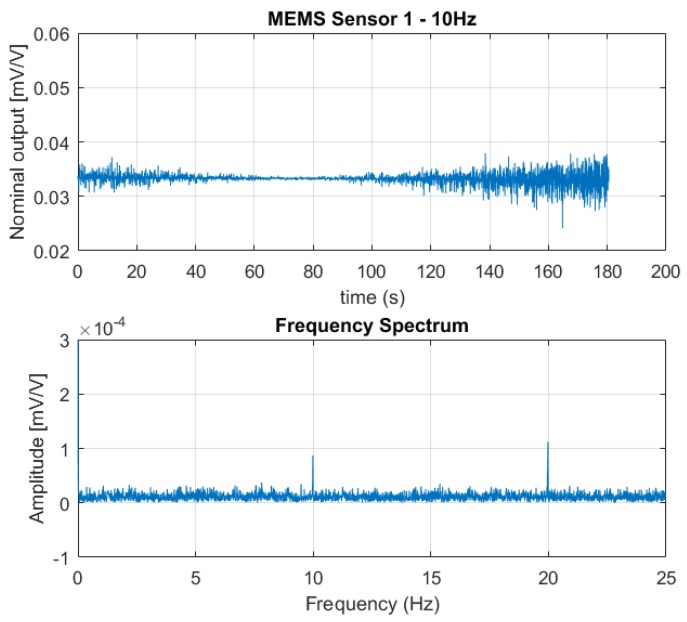
The output signal from one of the sensors in the MEMS-based sensor system as a response to the 10 Hz excitation vibration (**above**) together with the fast Fourier transform of the data set (**below**). The fast Fourier Transform clearly shows the fundamental excitation frequency at 10 Hz with an amplitude far above the noise floor; also, the second order harmonics at 20 Hz is visible.

**Figure 8:**
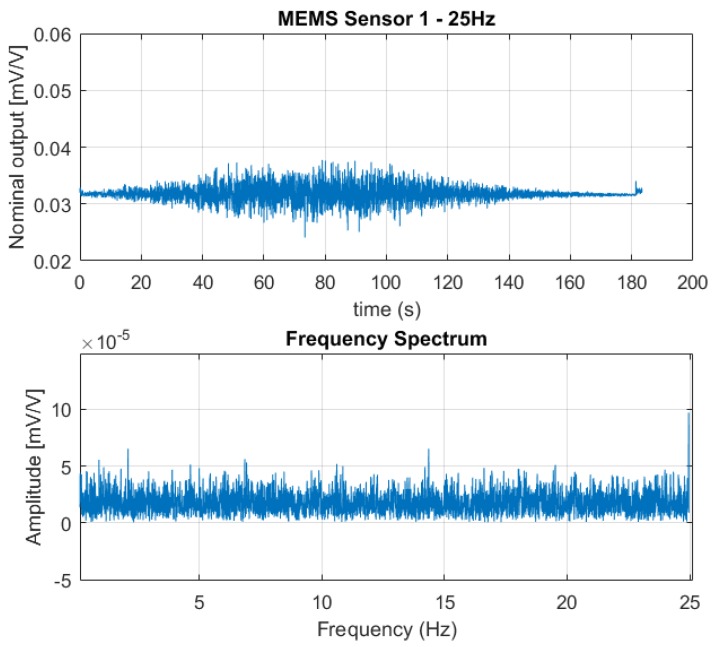
The output signal from one of the sensors in the MEMS-based sensor system as a response to the 25 Hz excitation vibration (**above**) together with the fast Fourier transform of the data set (**below**). The fast Fourier transform indicates the fundamental excitation frequency at 25 Hz, with an amplitude well above the noise floor. As the sampling frequency of the SDL is 50 Hz, we only have data points from the fast Fourier transform up to 25 Hz.

Figure 6, Figure 7 and Figure 8 show the output from one of the two MEMS-based sensor probes as a response to the 5 Hz, 10 Hz, and 25 Hz excitation vibrations. In the lower part of the figures, a fast Fourier transform (fft) of the data sets are shown. From the fast Fourier transform, it is clear that the sensors picked up rapid pressure pulses. The first (fundamental) excitation frequencies at 5 Hz, 10 Hz, and 25 Hz were detected with amplitudes well above the noise floor. The higher frequency peaks also present in the plots, are the higher order harmonics of the fundamental. For the 25 Hz excitation frequency, we are at the limit of what can be correctly captured. As a skilled reader would know, the Nyquist-Shannon theorem states that to be able to capture all the information in a bandlimited continuous time signal, the sample rate must be at least twice as fast as the maximum frequency of the time-signal [15].

The MEMS-based sensor system worked well also after the vibration test was completed, including enforcing the higher frequencies of 50 Hz and 70 Hz to the system. Therefore, the system robustness to shaking appeared to be good.

### 3.2. The Conventional Method

The conventional setup with the MMS cystometry equipment was not able to detect any rapid pressure pulses from the setup. However, normal functioning was verified after completing the vibration tests by applying an external static pressure. This result indicates that the pressure-transmission through the water-filled lines were too slow to be detected by the transducers.

## 4. Discussion

In our study, we compared the characteristics of a novel cystometry system using in-target organ sensor with a conventional system using water-filled lines. While the MEMS-based sensor system recorded real changes in applied pressure for excitation frequencies ranging from 5 Hz to 25 Hz, such small and high-frequency stimuli were not transmitted through the water-filled lines connected to the external transducers.

There is an obvious difference between the sensor systems: The in-target organ sensor is a microsensor with minute mass displacement versus approximately 2.5 m of water-filled line. Even though the water-filled line is pressurized to 300 mg Hg to improve pressure transmission, small pressure changes with frequencies of 5 Hz or above cannot be transmitted. The study was done with the length of water-filled lines as provided for clinical use by the supplier. Shorter lines could be expected to result in improved frequency response and should be addressed in future studies.

The urinary bladder is located inside the abdominal wall. The pressure during a bladder contraction results in modest increases, physiologically in the range 0–40 mbar. However, pressure is also exerted by the abdominal wall by movement or muscle contraction. To determine the detrusor pressure (*P_det_*), i.e., the pressure exerted by the bladder itself, the abdominal pressure needs to be subtracted from the pressure recorded inside the bladder:

(1) $$P_{det}=P_{ves}-P_{abd}$$

**Figure 9:**
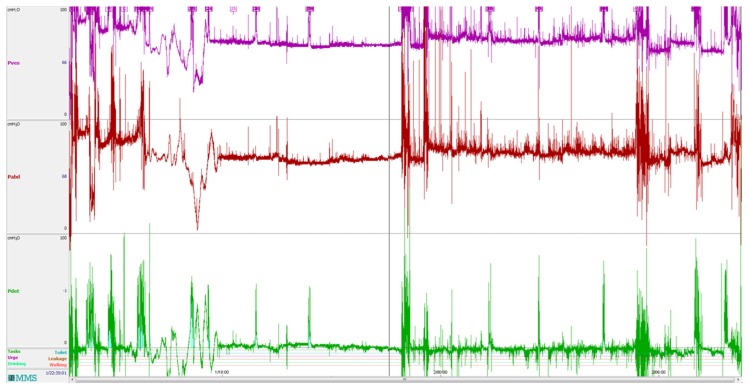
A typical cystometry curve from ambulatory urodynamics using a portable data logger from Medical Measurement Systems (Enschede, The Netherlands). The unit along the y-axis is cmH_2_O, the unit along the x-axis is the time of day (in hours). Bladder pressure (*P_ves_*) is shown in the upper part and reference abdominal pressure (*P_abd_*) in the middle. The lower curve is the calculated detrusor pressure (*P_det_*). Detrusor activity is difficult to identify, even in an expanded graph.

Figure 9 shows a curve during cystometry with all three parameters. In clinical practice, abdominal pressure is often recorded from the rectum. This is part of the gastrointestinal system, and in itself, a contractile organ. In many cases, this will cause significant artefacts in the determination of detrusor pressure. There are several devices on the market for ambulatory cystometry. According to our experience at the clinic, there are important limitations with this equipment when connected to water-filled lines. As shown in Figure 9 the cystometry curve is difficult to interpret, even in an expanded graph, due to movement artefacts. This illustrates the fact that physiological characteristics of the target organ are not recorded, but the patient’s activities are.

According to the standards of International Continence Society (ICS), cystometry can be done with water-filled lines connected to an external pressure transducer [7]. However, the importance of avoiding errors when using water-filled lines is well recognized [16] and regarded as critical for patients by ICS’s basic training program [8]. ICS acknowledges new microtip catheters and air-filled lines, and the fact that these methods have the advantage of no bubbles in the fluid system. Furthermore, that the results obtained from the different methods are not entirely identical. The standards, however, do not elaborate on other important aspects that may also influence the measurements.

A superior solution to avoid measurement artefacts is to place the abdominal (or reference) sensor into inert tissue in the prevesical space close to the bladder sensor. This will eliminate any artefact through smooth-muscle activity. However, the sensor must be inserted through the skin and into tissue inside the abdominal wall.

With an optimal placement, it is also crucial to have a sufficient synchronization of data sampling to obtain a correct real-time value. Movement or contraction of the abdominal muscles may cause rapid changes. Any delay in the sampling of the reference pressure could cause artefacts. Currently, a sampling rate of 8 Hz is standard in cystometry. However, achieving high-quality and high-resolution measurements may require far higher sampling rates. The technical and clinical implications of higher sampling rates need further studies.

Laws of fluid dynamics describe pressure transmission through a water-filled line. It is not within the scope of this paper to discuss a precise mathematical model, but rather to present some of the sources of error. Poiseuille’s equation gives the pressure drop (Δ*P*) in an incompressible fluid through a long cylindrical pipe of constant cross-section:

(2) $$\Delta P=\frac{8\mu LQ}{\pi R^{4}}$$

where *L* is the length of pipe, *μ* is the the dynamic viscosity, *Q* is the volumetric flow rate, and *R* is the pipe radius. The equation is valid for laminar flow, which holds in our case with repeated flushing of the lines with approximately 2–3 mL/min and a rough estimate of Reynolds number below 100 (for the interested reader, we recommend further reading in scholarly articles on the science of fluid dynamics). In principle, the pressure-change from the target organ to the external transducer will depend on the length and radius of the line, as obvious from Equation (2). Thus theoretically, when length and radius of line approximates a critical unit, the external transducer will no longer be able to detect any pressure change. An air-filled catheter has been developed, which reduce dynamic viscosity significantly, but on the other side reduces transmission being a compressible substance.

Movement may cause artefact in water-filled lines through different mechanisms. Both gravity and horizontal movement in a mobile patient will induce acceleration or deceleration in the water column. The pressure exerted on the external transducer through these mechanisms will depend on the acceleration and mass of water in the line. Thus, the longer and the larger radius of line, the more errors.

The lines are made from flexible material, usually medical grade plastics. Elasticity in the lines will, to some extent, also dampen the pressure transmission. An elastic line will cause a higher loss of pressure from the target organ to external transducer than a stiffer. However, highly rigid lines will never be feasible in a clinical setting.

Bending and twisting the lines during movement will also cause errors. Firstly, this will change the diameter of the line and cause a loss of transmission from the target organ to the external transducer. In the extreme case of a complete bending of the line, pressure transmission will cease completely. Pressing the line will also cause displacement of water and an erroneous signal.

In our opinion, there are several practical and theoretically limitations in pressure measurement systems using water-filled lines. As our experiments demonstrate, in-target organ sensors have superior properties to measure high-frequency and low-pressure stimuli, which is an important feature for cancelling of measurements not being related to pressure changes but to movements. This will be a requirement to proceed with long-term implantable pressure measurement systems in the future.

In an ideal setting, measurements should be done non-invasively with small portable equipment, leaving the individual completely physically and mentally unaffected. However, non-invasive measurements often have low precision and many sources of error. Thus, obtaining high-quality measurements of pressure involves invasive procedures.

## 5. Conclusions

Pressure is a vital parameter for the performance of organs and the health in humans. In medical practice and research, high-quality and high-resolution measurements are essential for diagnostics and therapeutic purposes. In organs like the circulatory system and the urinary bladder, water-filled lines connected to externally located transducers have been recommended and in use for decades. Recent advances in MEMS technology offer minute pressure sensors with novel properties. We conclude that there are both theoretical and practical limitations to measurements done with water-filled lines connected to a transducer. An in-target organ pressure sensor is superior in detecting minute pressure pulses with high frequency. This solution is promising for improving the quality of measurements and the option of long-term implantable devices. Further studies are needed to assess the clinical value of an improved measurement quality. In the future, implanted devices may develop new health services with home-monitoring and alarm systems to improve patients’ empowerment and quality of life.
